# T Cell-Engaging Bispecific Antibodies Targeting gp100 and PRAME: Expanding Application from Uveal Melanoma to Cutaneous Melanoma

**DOI:** 10.3390/pharmaceutics16081046

**Published:** 2024-08-06

**Authors:** Robin Reschke, Alexander H. Enk, Jessica C. Hassel

**Affiliations:** 1 Department of Dermatology and National Center for Tumor Diseases, University Hospital Heidelberg, 69120 Heidelberg, Germany; 2 German Cancer Consortium (DKTK), Core Center Heidelberg, 69120 Heidelberg, Germany; 3 German Cancer Research Center (DKFZ), 69120 Heidelberg, Germany

**Keywords:** tebentafusp, T cell-engaging bispecific antibody, ImmTAC

## Abstract

Uveal melanoma represents a rare and aggressive subtype of melanoma with limited treatment options and poor prognosis, especially in the metastatic setting. Tebentafusp, a bispecific fusion protein, offers a promising therapeutic approach by targeting gp100, an antigen highly expressed in uveal melanoma cells, and redirecting T cell-mediated cytotoxicity towards tumor cells. This review provides an overview of the preclinical and clinical data on tebentafusp in the management of metastatic uveal melanoma. We summarize the mechanism of action, clinical efficacy, safety profile, and ongoing research efforts surrounding this innovative immunotherapy. Preclinical studies have demonstrated the ability of tebentafusp to induce potent and specific anti-tumor immune responses against gp100-expressing uveal melanoma cells. Clinical trials have shown encouraging results, with tebentafusp exhibiting meaningful clinical activity in a subset of patients with metastatic uveal melanoma. Importantly, tebentafusp has also demonstrated a manageable safety profile. By specifically targeting tumor cells expressing gp100, tebentafusp offers a promising therapeutic avenue for individuals with metastatic uveal melanoma, meeting a significant clinical need in this context. Continued clinical trials will provide additional insights into the impact of tebentafusp on treatment-resistant metastatic cutaneous melanoma. Furthermore, we are exploring the potential of T cell engagers directed against the cancer testis antigen PRAME, which could have widespread utility in the treatment of cutaneous melanoma as well as other PRAME-expressing malignancies.

## 1. Introduction

**Figure 1:**
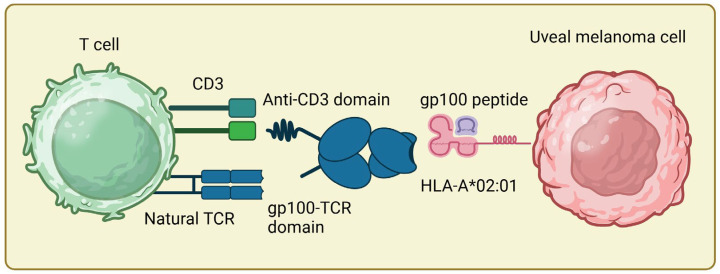
Tebentafusp is a so-called bispecific T cell engager, which recognizes an HLA-A*02:01-presented gp100 peptide with a high-affinity soluble TCR and binds to T cells with the effector domain, an anti-CD3 antibody, activating them regardless of their intrinsic specificity. Thus, it mimics the immune synapse between T cell and cancer cell. Tebentafusp thereby leads to polyclonal activation of T cells in the tumor environment, followed by cytokine release and tumor defense (figure created with BioRender).

Immunotherapy with immune checkpoint inhibitors (ICI) has changed the treatment landscape of many solid tumors. However, tumors exist that do not profit from ICI. Uveal melanoma, or choroidal melanoma, is a rare variant of melanoma that does not originate from the skin but from the melanocytes in the eye. In most cases, melanocytes of the choroid are involved, but the iris or ciliary body can also be affected. Uveal melanoma frequently leads to distant metastases, primarily in the liver (90%) [1,2]. Unlike cutaneous melanomas, uveal melanoma responds poorly to checkpoint inhibitor therapy; this is one reason for a significantly worse prognosis, with patients typically experiencing a median survival time of approximately one year following the detection of metastases [3]. Despite recent advancements in immune checkpoint blockade in cutaneous melanoma and other solid tumors, outcomes for these patients have remained stagnant for decades, with no notable improvement. With tebentafusp, the first immunotherapy has been approved for metastatic uveal melanoma, which significantly improves overall survival [3]. Tebentafusp is a bispecific protein from the group of so-called T cell engagers or immune-mobilizing monoclonal TCRs against cancer (ImmTACs), a new form of immunotherapy [4]. It possesses a highly specific T cell receptor that recognizes an HLA-A0201-presented peptide of the melanocytic differentiation antigen glycoprotein 100 (gp100), which is expressed in normal melanocytes as well as melanoma cells, and is fused to a CD3 antibody that binds and activates T cells [4]. However, the T cell receptor was designed to only recognize the gp100 peptide presented by the specific genetically determined surface marker HLA-A*02:01. This human major histocompatibility complex (MHC-class I) is the most common type (approximately 50%) in Caucasians and, therefore, enables approximately half of patients with advanced uveal melanoma to undergo treatment with tebentafusp [5]. CD4^+^/CD8^+^ T cells activated in this manner and bound to the tumor cells induce cell death in uveal or cutaneous melanoma cells [6,7] (Figure 1). Its mechanism of action underlines the potential of bispecific antibodies to leverage the body’s immune system in targeting and eliminating cancer cells, enabling more effective and personalized cancer therapies. The application of bispecific T cell engagers (BiTEs) in cancer therapy is not limited to uveal melanoma. Treatment with bispecific T cell engagers (BiTes) is based on a two-directional antibody with two different cell targets [8]. One end binds to the tumor-associated antigen, and the other binds to a CD3 of the T cell. This dual targeting results in an “immune synapse”, which enables the activation and cytotoxic response of T cells against the cancer cells [8]. Various bispecific antibody constructs are being developed and tested for a range of malignancies, including hematologic cancers, such as leukemia and lymphoma, as well as solid tumors, such as gastrointestinal tumors, and breast and lung cancer [9,10]. Clinical trials have shown encouraging results, demonstrating significant anti-tumor activity and manageable safety profiles. Despite their promise, challenges remain, such as optimizing the stability and half-life of these molecules, managing potential immune-related side effects, and ensuring effective tumor penetration. Ongoing research aims to address these issues and enhance the therapeutic efficacy of bispecific antibodies across cancers. In summary, T cell-engaging bispecific antibodies represent a novel approach in cancer therapy, leveraging the power of the immune system to provide more targeted treatment options for patients with various types of cancer, even those refractory to standard therapies. Tebentafusp, for instance, shows efficacy in the treatment of uveal melanoma, which is usually refractory to ICI. Bispecific antibodies might also work in less immunogenic tumor microenvironments and should be tested on other rare, aggressive entities.

## 2. Immunological Effects of Tebentafusp

**Figure 2:**
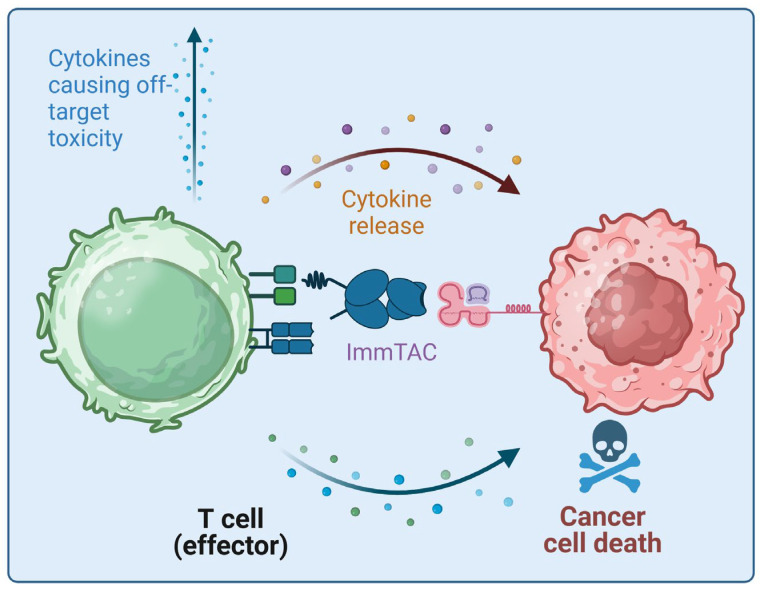
This schematic illustrates how an ImmTAC links T cells and tumor cells and creates an “immune-synapse”, resulting in T cell activation and cytokine release. This process ultimately leads to tumor cell death. However, the released cytokines can also cause adverse events, such as a collateral effect (figure created with BioRender).

Tebentafusp has an anti-CD3 single-chain variable fragment (scFv) which binds and activates CD3^+^ T cells [11]. This domain is connected via a linker to the soluble TCR domain, which recognizes specific peptides presented by human leukocyte antigen (HLA) complexes. In addition, a disulfide bridge stabilizes the soluble TCR [11]. Interestingly, ImmTACs can target intracellular epitopes, which make up the majority of neo-antigens [11]. ImmTAC TCRs have higher affinity than normal TCRs to enhance successful binding to the target [11]. Tebentafusp targets the melanoma-specific antigen gp100 with a T cell receptor arm, which recognizes HLA-A*02:01-gp100 peptide complexes presented on the tumor cell surface [12]. Thereby, T cells in the tumor microenvironment are bound to the tumor cells and activated independent of their specificity, resulting in tumor cell killing and the secretion of cytokines [12] (Figure 2). 

It also seems that neighboring tumor cells are lysed as a result of the cytokines being released [8]. It is hypothesized that the achieved tumor cell killing might trigger “epitope spreading” and additional T cell activation and the upregulation of checkpoint molecules in the tumor microenvironment [11]. Cytokines such as IFN-γ, TNFα, IL-2, and IL-10 were increased significantly 8–24 h after tebentafusp infusion [7], indicating a temporary effect on the immune system within the first day. When the serum samples of patients treated with tebentafusp were analyzed, the most upregulated inflammatory markers after the first dose of tebentafusp were the IFN-inducible chemokine CXCL10 and the cytokine IL-6 [7]. Interestingly, high levels of CXCL10 correlated with survival in tebentafusp-treated patients (uveal melanoma and cutaneous melanoma) [7]. It has been reported that CXCL10 is able to recruit additional CD8^+^ T cells from circulation to the tumor microenvironment via a chemokine gradient, resulting in CXCL10 being a good prognostic marker in patients with metastatic, cutaneous, or uveal melanoma treated with immune checkpoint blockade or tebentafusp [7,13]. In patients treated with tebentafusp, a reduction of circulating CD8 T cells, in particular effector memory T cells, expressing the cognate receptor CXCR3 was observed, while an increase in cytotoxic T cells in the tumor microenvironment was measured [7]. High levels of IL-6 can cause systemic immune dysfunction [14]. CXCL10 and IL6 have also been implicated in the immune pathology of immune-related adverse events [15,16]. Cutaneous and gastrointestinal irAEs can be mediated by IFN-γ-producing tissue-resident memory T cells, causing the downstream production of CXCL10 [15,17]. It has been shown that rashes occur because cytotoxic T cells also target regular skin melanocytes expressing gp100 [7,18,19]. Hypopigmentation or disappearing nevi in uveal melanoma patients treated with tebentafusp might be explained similarly. Early rash after tebentafusp treatment did not correlate with an OS advantage [20]. For cutaneous melanoma tumors, intrinsic biomarkers, such as a 7-marker signature, consisting of Bax, Bcl-X, PTEN, COX-2, β-Catenin, MTAP, and CD20, exist, stratifying the risk of progression [21,22]. Other biomarkers are more oriented towards immune-cell recruitment and helping to guide immunotherapy in advanced cutaneous melanomas [13]. In uveal melanoma biomarkers, stratifying progression or guiding therapy with tebentafusp are currently lacking. Circulating tumor DNA (ctDNA) is currently evaluated as a promising biomarker. However, further research in this area needs to be conducted.

## 3. Updates on Tebentafusp in Uveal Melanoma

In the phase 3 study that led to the approval of tebentafusp for advanced uveal melanoma, 378 patients with metastatic uveal melanoma were randomized in a 2:1 ratio. Patients were stratified by LDH levels to receive either tebentafusp in a dose-escalation regimen of 20 μg to 68 μg weekly, intravenously, or investigator’s choice (IC) therapy with pembrolizumab 2 mg/kg every three weeks, ipilimumab 3 mg/kg four times at three-week intervals, or dacarbazine (DTIC) 1000 mg/m^2^ every three weeks [20]. Patients eligible for this study had metastatic uveal melanoma and matched HLA-A*02:01 status, with no prior systemic therapy or liver-directed therapy (except surgery). The initial analysis of the study showed a significantly improved overall survival time for patients receiving tebentafusp compared to IC [20]. In the IC arm, most patients, namely 82%, received pembrolizumab therapy. In the recently published 3-year follow-up of the study, the benefit for overall survival time in tebentafusp-treated patients was confirmed [23]. At 36 months, 27% of patients treated with tebentafusp and 18% of patients in the control arm were still alive [23]. The median overall survival time was 21.6 months in the tebentafusp group and 16.9 months in the control group (hazard ratio, 0.68; 95% confidence interval, 0.54 to 0.87). A notable feature of tebentafusp is that this overall survival advantage persists despite having only a minimal impact on progression-free survival. The median progression-free survival time in the tebentafusp cohort was 3.4 months compared to 2.9 months in the control arm [23]. One reason for this is that even patients who show progression as their best response according to RECIST (Response Evaluation Criteria In Solid Tumors) can still benefit from tebentafusp in terms of overall survival (HR 0.62 compared to IC; 95% CI 0.44–0.89) [23]. A better predictor of overall survival and the effectiveness of tebentafusp than RECIST response seems to be the decrease in ctDNA from baseline to week 9 of first-line tebentafusp therapy [4,23]. Unfortunately, the ability to determine ctDNA in clinical practice is not yet available, so we continue treatment beyond progression until the tumor burden significantly increases. Presumably, tebentafusp prolongs survival in progressive patients by slowing progression. However, the phase 3 study was criticized for not having combination immunotherapy with ipilimumab and nivolumab in the control arm. A propensity score analysis using the data of tebentafusp and pembrolizumab therapy from the first-line trial and ipilimumab + nivolumab from a Spanish phase II study (NCT02626962) showed an advantage for tebentafusp versus combination immunotherapy with nivolumab and ipilimumab in metastatic uveal melanoma [24]. Surprisingly, patients initially unresponsive to tebentafusp have shown significant responses to immune checkpoint inhibitors, suggesting potential co-therapy advantages [25].

According to the approval study, side effects typically occur early in the treatment phase, especially with the first three doses (Table 1) [20,23], which is why tebentafusp is dosed gradually. The first three doses are administered in a hospital setting (with intensive-care backup) to detect any side effects as early as possible. 

## 4. Adverse Events of Tebentafusp

Tebentafusp can cause a range of side effects. These side effects can vary in severity and are primarily related to the immune activation that tebentafusp induces. The side effects of tebentafusp can be divided into two groups, which can be explained pathophysiologically. On the one hand, cytokine release-related side effects occur, with the main symptoms being fever (76%), chills (49%), and hypotension (38%); on the other hand, T cell activation targeting gp100-expressing skin melanocytes causes skin-related adverse events such as rash (83%) and pruritus (70%) [27]. Cytokine release syndrome can be recognized easily and, if necessary, treated, although this is rarely severe (1% grade 3/4) [23]. The proportion of treatment discontinuations due to side effects overall was low in the randomized clinical trial (2% tebentafusp group, 5% control group) [23]. Published practical guidelines for managing adverse events associated with the T cell engager bispecific tebentafusp could serve as a valuable resource for addressing symptoms of cytokine release syndrome in patients undergoing treatment with other BiTes such as PRAME-targeted T cell engagers [28].

Cytokine release syndrome (CRS) is one of the most common side effects associated with tebentafusp and results from the rapid release of cytokines, such as IL-6, shortly after infusion [28]. In rare cases, CRS can be life-threatening, necessitating close monitoring in an experienced in-patient ward and management. As previously described, tebentafusp can also result in various dermatologic side effects, including rash, erythema, pruritus, and cutaneous oedema [28]. These skin reactions are generally mild to moderate in severity and often resolve by themselves. If more severe, they can be managed with topical corticosteroids [28]. Antihistamines could be used for pruritus. Fatigue is a common side effect of tebentafusp and other immunotherapies. Other reported symptoms are gastrointestinal issues such as nausea or hepatotoxicity shown by elevated aspartate aminotransferase/alanine aminotransferase (AST/ALT) levels and/or bilirubin levels [23,28]. To prevent adverse events such as hypotension, intravenous fluids are given prior to the administration of tebentafusp [28]. Antipyretics can be given to mitigate fevers and can be administered prophylactically [28]. Infusion reactions observed in other immunotherapies are only rarely seen with tebentafusp [20]. Patients with prior cardiac disease should receive a cardiologic assessment, including an electrocardiogram (ECG) and echocardiography, and treatment initiation should be discussed depending on the results [28]. CRS symptoms might become more likely in patients with a history of cardiac disease [29]. In summary, while tebentafusp offers a promising therapeutic option for patients with metastatic uveal melanoma, it can be associated with a range of side effects primarily due to its mechanism of action involving robust immune activation. Thus, initiation of tebentafusp treatment (first three cycles) should be carried out with intensive care backup for the rare occurrence of severe CRS [28]. Corticosteroids such as dexamethasone could be considered when patients have experienced grade 2 CRS during a previous cycle [28]. The anti-IL-6 receptor antibody tocilizumab should be used for grade 3 CRS [28,30]. Subsequent cycles can be administered in an outpatient ward with the required expertise, such as a dermato-oncological outpatient ward. If these prerequisites are followed, adverse events caused by tebentafusp are manageable. They can be treated and resolved quickly in most cases. 

## 5. T Cell-Engaging Bispecific Antibodies in Cutaneous Melanoma and Other Solid Tumors

Tebentafusp has also undergone evaluation in the context of metastatic cutaneous melanoma. An initial study in 2020 demonstrated clinical activity in patients with HLA-A*02:01+ cutaneous melanoma refractory to standard immunotherapy [7]. Thus, T cell-engaging bispecific antibodies hold the potential to also treat cutaneous melanoma patients and potentially other solid tumors expressing relevant tumor antigens. It is important to note, however, that therapy with bispecific antibodies can lead to the expression of immune checkpoint molecules such as PD-1 or CTLA-4 [9]. This T cell exhaustion can lead to treatment resistance. A strategy to counteract this exhausted phenotype could be to combine therapy with immune checkpoint inhibitors [12]. Tebentafusp might also lead to more relevant cytotoxic T cells in the tumor microenvironment, further facilitating the effects of ICI. Recently, promising results were observed with the combination of tebentafusp and durvalumab (anti-PD-L1) and/or tremelimumab (anti-CTLA-4) in individuals with metastatic cutaneous melanoma, especially among heavily pretreated, ICI-refractory patients [29]. This phase Ib trial included eighty-five patients and reported no new adverse events or treatment-related deaths [29]. Within this heavily pretreated cohort, the response rate was 14%. For 41% of the patients, tumor shrinkage was documented. The median overall survival time was 18.7 months. Other ongoing clinical trials are currently evaluating the efficacy of tebentafusp in patients with treatment-refractory metastatic cutaneous melanoma or those with cutaneous melanoma exhibiting molecular relapsed disease (NCT05315258, NCT05549297). A few other antigens, such as CSPG4 (glycoprotein and chondroitin sulfate proteoglycan), MC1R (melanocortin receptor group), DR5 (death receptor 5), and MSCP (glycoprotein and chondroitin sulfate proteoglycan) are currently being explored as targets for bispecific antibodies treating melanoma [12]. CSPG4, for instance, is only expressed in melanoma and not in healthy tissue [12]. Additionally, clinical testing of a novel target for T cell-engaging bispecific antibodies, known as PRAME, has commenced. PRAME stands out as one of the most widely expressed cancer-testis antigens [31]. High expression of PRAME has been identified in various gynecologic tumors, including endometrial carcinomas (82%), uterine serous carcinomas (82%), ovarian clear cell carcinomas (90%), as well as dermatologic tumors such as basal cell carcinomas (62%), primary and metastatic melanoma (80–90%), or uveal melanoma (26–45%) [32,33,34]. PRAME is prominently expressed in hematopoietic malignancies as well, with notable prevalence in conditions such as acute myeloid leukemia (AML) (40–60%), acute lymphoblastic leukemia (ALL) (20–40%), myeloma (20–50% of cases), and chronic myeloid leukemia (CML) (30–40%) [32]. PRAME expression extends to various other cancer types, including uveal melanoma, non-small cell lung cancer (NSCLC), kidney cancer, bladder cancer, head and neck squamous cell carcinoma (HNSCC), and esophageal carcinoma. Additionally, its presence has been associated with adverse prognosis in breast cancer and neuroblastoma [32]. T cell engagers targeting PRAME are presently undergoing extensive evaluation in large phase 1 and 2 clinical trials for all recurrent or refractory PRAME-positive solid tumors, encompassing cutaneous melanoma (NCT05958121 and NCT04262466). The first results of the phase 1 trial on metastatic melanoma have been presented at ASCO this year. It was reported that IMC-F106C demonstrated clinical activity in PRAME+ ICI-pretreated cutaneous melanoma patients without other clinical options, resulting in longer PFS and OS [35]. A phase 3 trial of IMC-F106C in combination with nivolumab as a first-line therapy for metastatic melanoma has been initiated (PRISM-MEL301; NCT06112314). These studies are restricted to patients with HLA-A*02:01+ (Table 2). As PRAME is expressed not only in solid tumors but also in the cells of leukemia and myeloma patients, this suggests a broad potential application [36]. Toxicity associated with its mechanism is similar to tebentafusp with the exception of rash, which is much less frequent; this is possibly because PRAME is not expressed, or only weakly expressed, in normal skin melanocytes. Preliminary results of another PRAME-ImmTAC indicated mild cytokine release syndrome symptoms in most patients. One instance of grade 4 cytokine release syndrome (CRS) and neurotoxicity was noted for IMA203CD8 GEN2 [37]. Taken together, bispecific antibodies targeting tumor-specific antigens seem to have manageable adverse events.

## 6. Challenges and Future Directions

At this point, therapy with ImmTACs is mostly limited to HLA-restricted antigen presentation. Since only about half of the population has this HLA phenotype, a future goal would be to extend treatment to patients with other HLA types. The approach of using ImmTACs to create a crucial “immune synapse” between T cells and tumor cells within the tumor microenvironment holds promise for a multitude of tumors. This is particularly compelling for immunosuppressive tumor microenvironments where tumor cells or recruited immunosuppressive bystander cells, such as myeloid cells, typically prevent T cells from becoming activated into cytotoxic T cells. A key challenge, however, is recruiting sufficient T cells into the tumor microenvironment initially. In this context, chemokines might play a significant role in future therapeutic strategies [13]. An intriguing approach to achieving an optimal “hot” tumor condition could involve combinatorial therapies, such as a combination of ImmTACs with immune checkpoint inhibitors (ICI), targeted therapies, or novel approaches like STING agonists. Early successes with tebentafusp in uveal melanoma suggest that bispecific antibodies could be broadly applicable to other tumor types. Since these bidirectional antibodies typically target tumor-specific antigens, the occurrence of adverse events is usually limited. Cytokine-release symptoms often occur early in the first treatment cycles, necessitating monitoring in an experienced ward with ICU backup. After the initial cycles, treatment can be continued in an experienced outpatient setting, making ongoing therapy more convenient for patients. One disadvantage of tebentafusp is the requirement of weekly infusions, as its effects on the immune system are not permanent. Research on the memory function and clonality of T cells activated by tebentafusp and other bispecific antibodies should be performed. The weekly regimen necessitates regular visits and lab work and incurs high costs for healthcare systems worldwide. The short half-life is not only a problem of tebentafusp but also of many other constructs, including first-generation BiTEs [40]. For example, blinatumomab has a half-life of 2 to 3 h only, potentially requiring continuous administration [40]. Attempts to prolong half-life through the improvement of pharmacokinetic properties should also be undertaken, potentially resulting in the need for fewer infusions and reduced hospitalization.

Ideally, these novel targeted therapies should also be made accessible to patients in less wealthy countries by reducing their costs eventually. Tebentafusp was the first immunotherapy for the rare cancer of uveal melanoma. Therapy with ImmTACs could be crucial for treating rare and aggressive cancers such as uveal melanoma that do not respond to ICI. Collaboration between centers is needed to conduct larger randomized trials and obtain approval for the treatment of these rare cancers as well. T cell-engaging bispecific antibodies should also be tested for ICI-refractory tumors as a second-line treatment approach.

## 7. Conclusions

In conclusion, tebentafusp represents the first immunotherapy available for patients with metastatic uveal melanoma, offering a significant survival advantage over other therapies, such as immune checkpoint inhibitors. However, it is essential that patients have the appropriate HLA type (HLA-A*02:01). Experience in administering the medication and a hospital setting with intensive care backup are necessary for therapy initiation in the clinical setting. Later on, after the first three treatment cycles, CRS symptoms only rarely occur. Tebentafusp is now also being tested in a randomized trial for patients with cutaneous melanoma refractory to anti-PD1 therapy (NCT05549297). In addition, current studies are focusing on developing additional targets for bispecific antibodies in the treatment of cutaneous melanoma, such as PRAME. Furthermore, mechanistic biomarkers are needed to guide T cell engager therapy in uveal and cutaneous melanoma and beyond. Larger studies are needed to test combinatorial therapies with ICI or BRAF/MEKi and evaluate the optimal dosage and sequencing of therapies in combination with bispecific antibodies. It remains to be seen if combination therapy is as well tolerated as ICI or BRAF/MEKi alone. In patients with cutaneous melanoma, we have reported a high satisfaction with the safety profile of these therapies, even in the adjuvant setting [41]. Considering the T cell exhaustion observed in patients treated with tebentafusp, this might lead to exploring other combination therapies in the future, such as bispecific antibodies combined with STING agonists [42]. Taken together, tebentafusp and other T cell-engaging bispecific antibodies hold the potential to expand efficacy to non-responder patients with cutaneous melanoma or other solid tumors. These novel approaches might turn an immune-deserted cold tumor into an inflamed tumor responsive to therapy. 

## Figures and Tables

**Table 1:** Most common treatment (Tx)-related adverse events [23]. Rash is a composite term for all skin-related adverse events (except pruritus). Cytokine release syndrome was graded according to 2019 American Society for Transplantation and Cellular Therapy consensus grading [26].

Most Common Tx-Related Adverse Events	
Any Grade	Grade 3/4
Cytokine release syndrome 89%	Cytokine release syndrome 1%
Rash 83%	Rash 19%
Pyrexia 76%	Pyrexia 5%
Pruritus 70%	Pruritus 5%
Chills 49%	Liver-function tests 6%
Nausea 45%	Hypertension 4%
Fatigue 42%	Lipase increased 4%
Hypotension 38%	Hypotension 4%

**Table 2:** Completed and ongoing trials with T cell-engaging bispecific antibodies targeting gp100 and PRAME in treatment of refractory/recurrent cutaneous melanoma and beyond; MRD = molecular relapsed disease, detected in molecular screening; ^†^ PRAME-positive solid tumors such as lung, ovarian, endometrial, melanoma, or breast cancer; ^#^ preliminary results reported in conferences or in press releases. / = unknown.

T Cell Engagers in Cutaneous Melanoma									
Trial	Agents	HLA Type	Target Antigen	2nd Medication	Phase	Tumor Type	Patients	Biomarker	Response
Middleton [7]	Tebentafusp	HLA-A*02:01+	gp100		1/2	Metastatic cutaneous melanoma	61	CXCL10, IL6	65% Overall survival rate
Hamid [29]	Tebentafusp	HLA-A*02:01+	gp100	Anti-CTLA4 ± anti-PDL1	1b	Metastatic cutaneous melanoma	85	/	/
**Ongoing Trials**									**Prelim. Results ^#^**
NCT05315258 TebeMRD	Tebentafusp	HLA-A*02:01+	gp100	/	2	Cutaneous melanoma with MRD	600	ctDNA	/
Immunocore TEBE-AM NCT05549297 [38]	Tebentafusp	HLA-A*02:01+	gp100	±Anti-PD1	2/3	Metastatic cutaneous melanoma	460	ctDNA	/
Immatics TCER NCT05958121 [39]	IMA402	HLA-A*02:01+	PRAME	/	1a, 1b, 2	Solid tumors ^†^	145	/	/
Immunocore IMC-F106C-101 NCT04262466 [31]	IMC-F106C	HLA-A*02:01+	PRAME	±Anti-PD1 or tebentafusp or anti-VEGF-A or chemotherapy or kinase inhibitors	1/2	Solid tumors ^†^	727	ctDNA	Metastatic melanoma: clinical benefit rate (CBR) of PR + SD was 61% (19/31) [35]
Immunocore IMC-F106C PRISM-MEL-301	IMC-F106C	HLA-A*02:01+	PRAME	±Anti-PD1 ±anti-LAG3	3	Metastatic cutaneous melanoma	680	/	/
